# Supplementary material for: Accumulation of mutations in antibody and CD8 T cell epitopes in a B cell depleted lymphoma patient with chronic SARS-CoV-2 infection
Source: Nat Commun. 2022 Sep 23;13:5586. doi: 10.1038/s41467-022-32772-5 (PMC9508331; doi:10.1038/s41467-022-32772-5)
Supplement: Supplementary file 1 — Supplementary Information [file 41467_2022_32772_MOESM1_ESM.pdf]

Supplementary Information for Khatamzas et al

Accumulation of mutations in antibody and CD8 T cell epitopes in a B-cell depleted lymphoma patient with chronic SARS-CoV-2 infection

Supplementary Figures

5	20	27	33	41	50	54	61	62	64	68	71	78	83	97	117	127	134	141	152	154	Nucleotide substitution	Gene	Aminoacid Substitution
0	0	0	0	0	0	0	0	0	0	0	0	0	0	39	63	42	8	5	0	0	**A2270G**	ORF1AB	K669E
100	100	100	100	100	100	100	100	100	100	100	100	100	100	100	100	100	100	100	100	100	**C3037T**	ORF1AB	F924F
0	0	0	0	0	76	54	87	99	97	96	98	95	97	99	99	99	96	99	99	98	**A4241G**	ORF1AB	I1326V
0	0	0	0	0	19	14	67	99	87	80	96	88	99	100	99	100	99	99	98	99	**C5144T**	ORF1AB	L1627L
100	100	100	100	100	100	100	100	100	100	99	100	100	100	100	100	100	99	100	95	99	**C5178T**	ORF1AB	T1638I
0	46	0	0	0	0	0	0	0	0	0	0	0	0	0	0	0	0	10	0	0	**G9479T**	ORF1AB	G3072C
0	0	0	0	0	0	0	0	0	0	0	0	0	0	0	0	0	0	63	90	93	**A9741G**	ORF1AB	N3159S
0	0	0	0	0	43	28	6	0	0	0	0	0	0	0	0	0	0	0	0	0	**C10078T**	ORF1AB	F3271F
0	0	0	0	0	0	0	0	0	0	0	0	0	0	0	0	0	87	57	93	99	**C10116T**	ORF1AB	T3284I
0	0	0	0	0	0	0	0	0	0	0	0	0	0	0	0	0	93	67	97	99	**T11059C**	ORF1AB	T3598T
0	0	0	0	0	0	0	0	0	0	0	0	0	0	0	0	0	90	55	95	99	**A11451G**	ORF1AB	Q3729R
0	0	64	17	0	0	0	0	0	0	0	0	0	0	0	0	0	0	0	0	0	**C11575T**	ORF1AB	F3770F
0	0	0	0	0	0	0	0	0	0	0	0	0	0	0	0	0	92	0	0	7	**T11751C**	ORF1AB	L3829P
0	0	0	0	0	0	0	0	0	0	0	0	0	0	0	0	0	0	53	90	91	**A11839G**	ORF1AB	V3858V
0	43	15	33	100	100	99	100	100	99	100	99	100	99	99	99	99	97	100	98	99	**G12566T**	ORF1AB	V4101F
0	0	0	0	0	0	0	0	0	0	0	0	0	0	99	100	100	99	100	98	100	**C12756T**	ORF1AB	T4164I
0	0	62	13	0	0	0	0	0	0	0	0	0	0	0	0	0	0	0	0	0	**C13019T**	ORF1AB	L4252L
9	18	12	27	99	99	100	99	100	99	99	99	100	99	100	99	99	96	100	99	99	**T14313C**	ORF1AB	D4683D
100	100	100	99	99	100	98	100	100	100	100	100	100	100	100	100	100	100	100	99	99	**C14408T**	ORF1AB	P4715L
0	45	0	0	0	0	0	0	0	0	0	0	0	0	0	0	0	0	0	0	0	**C14599T**	ORF1AB	L4779L
0	0	0	0	0	28	17	69	100	89	49	0	0	0	0	0	0	0	0	0	0	**C18981T**	ORF1AB	H6239H
0	0	0	0	0	0	0	0	0	0	0	0	0	0	0	50	23	0	0	0	0	**G19542A**	ORF1AB	M6426I
100	100	100	100	100	100	100	100	100	100	100	100	100	100	99	100	100	98	100	97	99	**G19825T**	ORF1AB	V6521L
0	0	0	0	0	35	43	0	98	82	78	100	75	99	100	100	100	90	100	100	100	**3del21990**	S	Y145del
0	0	0	0	0	0	0	0	0	0	0	0	0	0	0	0	0	0	55	91	93	**G22992A**	S	S477N
0	0	0	0	0	0	0	0	0	0	38	95	91	100	99	98	99	97	99	98	99	**G23012A**	S	E484K
100	100	100	100	100	99	100	100	100	100	100	100	100	100	100	100	100	100	100	99	100	**A23403G**	S	D614G
0	44	0	0	0	0	0	0	0	0	0	0	0	0	0	0	0	0	0	0	0	**C25702T**	ORF3A	P104S
0	0	0	0	0	0	0	0	0	0	0	0	0	0	0	67	51	6	5	0	0	**C27707T**	ORF7A	A105V
0	0	0	0	0	28	14	74	98	92	87	97	92	99	98	100	100	99	100	96	98	**T27793C**	ORF7B	F13S
0	0	0	0	0	0	0	0	0	0	0	0	0	0	0	0	0	0	0	40	14	**C28045T**	ORF8	A51V
100	100	100	100	100	100	100	100	100	100	100	100	100	100	100	100	100	100	100	100	99	**GGG28881AAC**	N	RG203KR
0	0	0	0	0	0	0	66	100	85	87	98	90	99	100	87	60	96	91	98	99	**C29247A**	N	T325K
0	47	19	35	100	100	99	99	100	98	99	99	100	99	100	98	100	97	100	98	99	**C29268T**	N	T332I

days post infection

Supplementary Fig. 1: Longitudinal SARS-CoV-2 sequence analysis. The heatmap shows the frequencies of mutations in coding regions for the 21 sequences of the patient in this study. Nucleotide and amino acid substitutions are indicated relative to the reference strain Wuhan-Hu-1. Six coding mutations were already present in the first sample at day 5, therefore likely representing the transmitted strain.

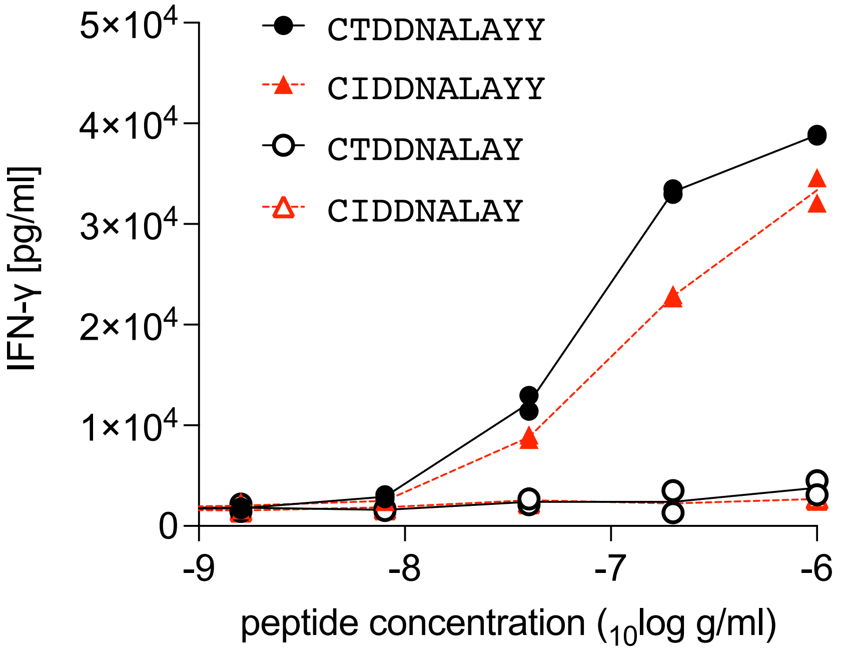

Supplementary Fig. 2: Reactivity of T cells to original and mutant epitope peptides. T-cell cultures were prepared from donor 3 by stimulation and expansion with the HLA-A*01:01-restricted epitope peptides CTDDNALAY(Y). Their reactivity to these epitopes and their mutant variants CIDDNALAY(Y) was tested by overnight co-culture with HLA-matched activated B cells and peptides at the indicated concentrations. Supernatants were harvested, and IFN-γ concentrations were determined by ELISA. Individual data points are shown with the line representing the mean.

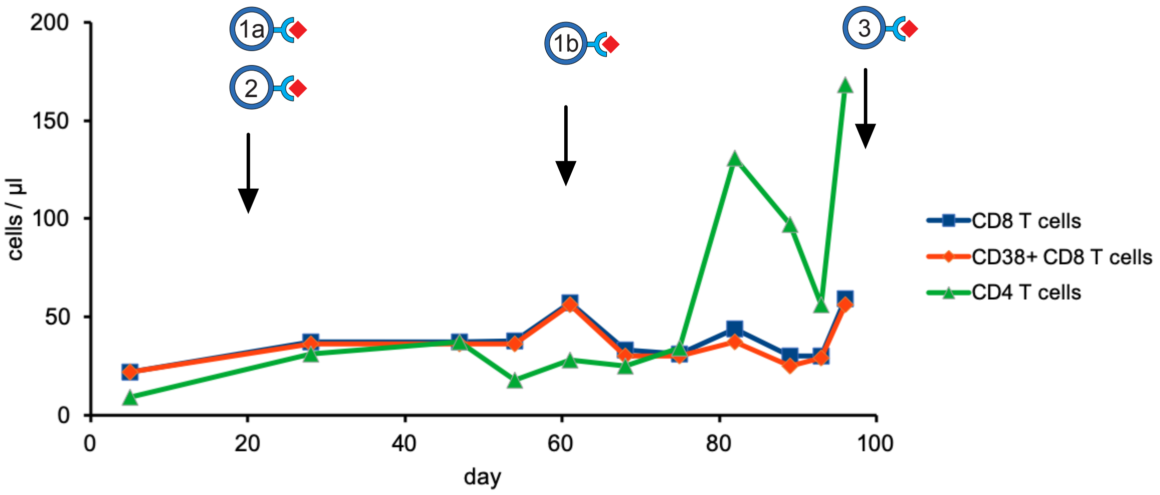

Supplementary Fig. 3: Absolute numbers of CD8 and CD4 T cells over time, and emergence of mutations in T-cell epitopes. Peripheral blood was obtained from the patient at the indicated times and analysed by flow cytometry. Analyses were not performed after day 96. Data on additional lymphocyte subsets are available in Supplementary Table 2.

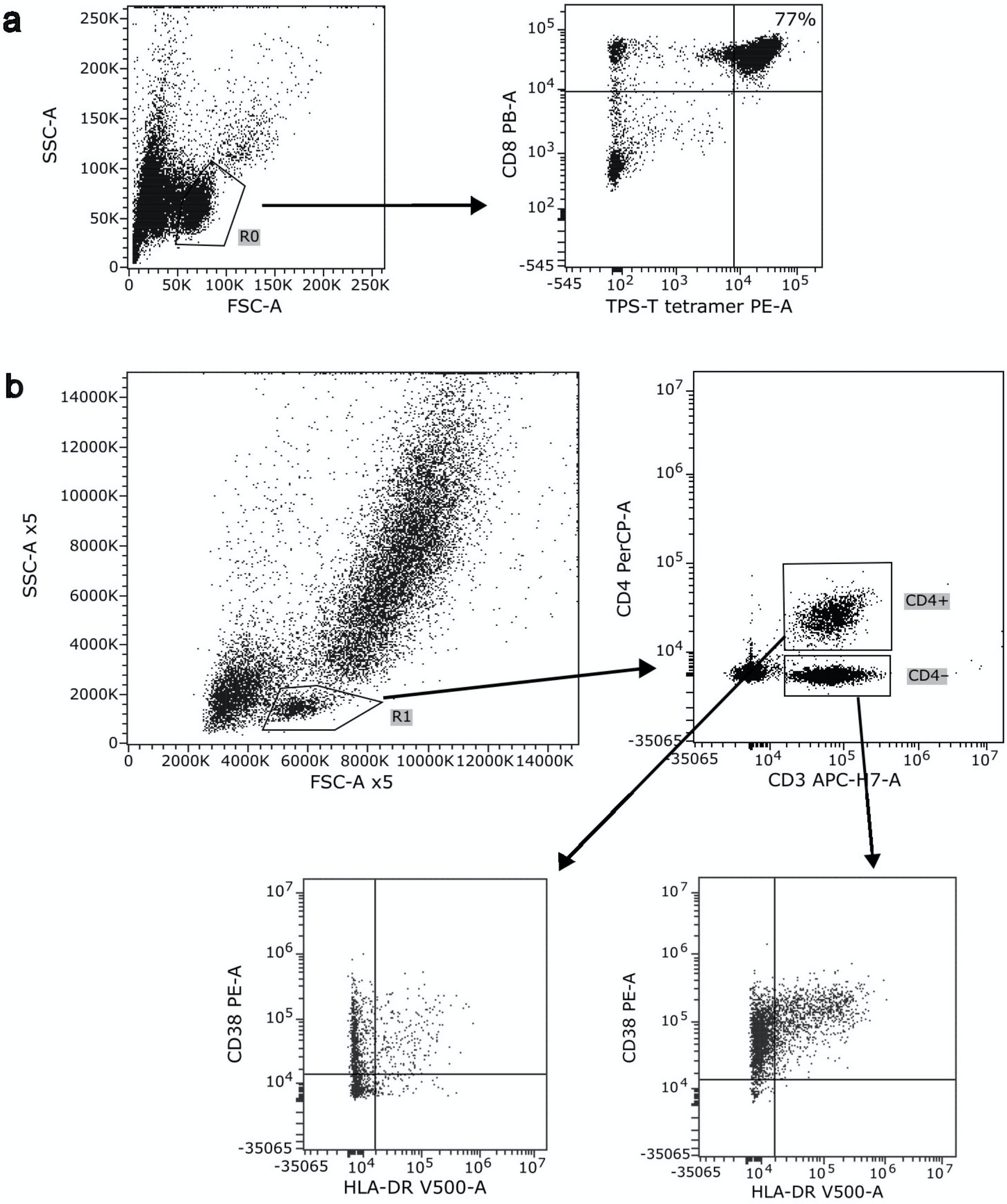

Supplementary Fig. 4: FACS gating strategy for tetramer analysis shown in Fig. 5 and 6 (a) and phenotypic analysis shown in Fig. 4 panel b (b). In (A), anti-CD8 was labeled with Pacific Blue; TPS-T, TPS-I, KPS-I represent PE-labeled HLA-B*35:01-peptide tetramers.

Supplementary Tables

	Reference range	d2	d10	d17	d26	d39	d52	d59	d68	d81	d88	d95
IgG	7.00 - 16.00 (g/l)	4.11	3.02	3.13	2.11	2.46	2.13	3.1	4.1	3.71	4.33	4.2
IgA	0.70 - 4.00 (g/l)	1.08	0.86	0.61	0.52	0.58	0.53	0.79	0.97	0.78	0.95	0.88
IgM	0.40 - 2.30 (g/l)	0.13	0.11	0.1	0.12	0.1	0.14	0.15	0.18	0.19	0.3	0.25

Supplementary Table 1: Immunoglobulin levels measured over time in the patient as part of clinical routine care. Values are in g/l, d indicates day of infection.

	Reference Range	d5	d28	d47	d54	d61	d68	d75	d82	d89	d93	d96
Lymphocytes absolute	1220-3560	74	121	266	170	286	264	326	452	315	252	452
% Leucocytes	18-46%	<1	2	1	1	2	2	2	4	2	4	7
T cells absolute (CD45+, CD3+)	620-2020	32	71	76	58	89	58	69	180	231	89	233
% Lymphocytes	60-85 %	43	59	29	34	31	22	21	4	41	35	52
CD4+ T cells absolute (CD45+, CD3+, CD4+, CD8-)	380-1300	9	31	37	18	28	25	34	131	97	56	168
% Lymphocytes	31-62 %	12	26	14	10	10	10	11	29	31	22	37
CD8+ T cells absolute (CD45+, CD3+, CD4-, CD8+)	160-810	22	37	37	38	57	33	31	44	30	30	59
% Lymphocytes	14-43%	30	31	14	22	20	12	9	10	9	12	139
CD38+ CD8 T cells absolute (CD45+, CD3+, CD4-, CD8+, CD38+)	≤ 210	22	36	36	36	56	30	30	37	25	29	56
% Lymphocytes	≤ 7%	30	30	14	21	20	11	9	8	8	12	12
HLA DR CD8 T cells absolute (CD45+, CD3+, CD4-, CD8+HLADR+)	≤ 124	19	31	25	30	40	18	16	19	16	17	39
% Lymphocytes	≤ 62%	84	83	68	79	71	54	53	43	55	59	66
CD4/CD8 Ratio	0.9-3.9	0.4	0.83	1	0.46	0.53	0.79	1.21	3.19	3.25	1.93	2.82
CD16+CD56+T cells absolute (CD45+, CD3+, CD16&56+)	≤ 210	2	4	5	5	11	3	3	14	9	2	9
% Lymphocytes	≤ 13%	3	3	2	2	3	1	1	3	2	8	2
B cells absolute (CD45+, CD19+)	70-420	0	0	0	0	0	0	0	0	0	0	0
% Lymphocytes	6-20 %	<1	0	0	0	0	0	0	0	0	0	0
NK cells absolute (CD45+, CD16&56+, CD3-)	50-510	42	50	190	113	195	208	256	272	186	161	222
% Lymphocytes	4-30%	56	42	71	67	68	79	79	60	59	64	49

Supplementary Table 2. Summary of lymphocyte subsets measured over time in the patient as part of routine clinical care. Peripheral blood mononuclear cells were immunophenotyped using standardised protocols. Absolute cell numbers are per µl. Source data are provided in the Source Data File.

Sample ID	Collection date	days after infection	mean coverage	coverage	GISAID ID
V2025779	11/05/2020	5	405	99.92	EPI_ISL_466909
DH206600	26/05/2020	20	1125	99.92	EPI_ISL_732538
DH206861	02/06/2020	27	1168	99.91	EPI_ISL_732539
DH207324	08/06/2020	33	602	99.89	EPI_ISL_732540
DH207597	16/06/2020	41	764	99.91	EPI_ISL_732537
DH207884	25/06/2020	50	410	99.92	EPI_ISL_732535
DH207990	29/06/2020	54	160	99.84	EPI_ISL_732536
V2074236	06/07/2020	61	814	98.86	EPI_ISL_1751601
DH208291	07/07/2020	62	307	99.8	EPI_ISL_732534
V2074567	09/07/2020	64	1320	98.96	EPI_ISL_1751602
V2074895	13/07/2020	68	1833	98.39	EPI_ISL_1751603
V2075248	16/07/2020	71	1745	98.98	EPI_ISL_1751604
V2075789	23/07/2020	78	1822	98.16	EPI_ISL_1751605
DH209138	28/07/2020	83	767	99.92	EPI_ISL_732531
DH209442	11/08/2020	97	1479	99.91	EPI_ISL_732532
V2080672	31/08/2020	117	1840	98.95	EPI_ISL_1751606
V2082641	10/09/2020	127	1920	96.91	EPI_ISL_1751607
V2083593	17/09/2020	134	1759	98.49	EPI_ISL_1751608
V2085389	24/09/2020	141	1834	98.17	EPI_ISL_1751611
V2088212	05/10/2020	152	956	98.67	EPI_ISL_1751623
DH211525	07/10/2020	154	3226	99.88	EPI_ISL_732658

Supplementary Table 3: Summary of sequence metadata with GISAID (https://www.gisaid.org) accession numbers. Source data are provided in the Source Data File.

	HLA-A		HLA-B		HLA-C	
Patient	A*01:01:01	A*02:01:01	B*08:01:01	B*35:01:01	C*04:01:01	C*07:01:01
HD1	A*03:01:01	A*32:01:01	B*15:01:01	B*35:01:01	C*03:03:01	C*04:01:01
HD2	A*01:01:01G	A*02:01:01G	B*08:01:01G	B*13:10	C*06:02:01G	C*07:01:01G
HD3	A*01:01:01G	A*24:02:01G	B*49:01:01G	B*55:01:01G	C*03:03:01G	C*07:01:01G
HD4	A*02:01:01G	A*11:01:01G	B*35:01:01G,	B*44:02:01G	C*04:01:01G	C*05:01:01G
HD5	A*02:01:01G	–	B*51:01:01G	–	C*14:02:01G	–

Supplementary Table 4. HLA class I type of the patient and five immune-competent convalescent donors (HD).

HLA allotype	anchor motif
A*01:01	X(ST)(DE)X_{5-7}Y
A*02:01	X(LIMv)X_{6-7}(LVIM)
B*08:01	XX(RK)X(RK)X_{2-3}(LVIM)
B*35:01	XPX_{5-8}(YFLVIM)
C*04:01	X(FYWX)(De)X_{4-6}(LFMivyw)
C*07:01	(KRx)(Rt)X_{5-7}(LFYMivw)

Supplementary Table 5. Anchor motifs used for epitope candidate identification for HLA class I allotypes of the patient. The one-letter amino acid code in uppercase or lowercase is used. X indicates any amino acid. Amino acids in brackets are alternatives; thus, (ST) indicates S or T. X_{2-3} indicates XX or XXX. Uppercase indicates most preferred amino acids, lowercase less preferred amino acids. A maximum of one less preferred anchor residue was admissible for an epitope candidate.

nt	aa ch.	day	protein	epitope candidates, HLA class I			
				A*01:01	A*02:01	B*35:01	C*04:01
4241	I1326V	50	nsp3	PTDNY**I**TTY	n	VPTDNY**I**TTY	PTDNY**I**TTY
9741	N3159S	141	nsp4	n	n	n	n
10116	T3284I	134	3CLpro	n	QV**T**CGTTTL	n	n
11451	Q3729R	134	nsp6	n	n	n	n
12566	V4101F	41	nsp8	n	ALWEIQQ**V**V	n	n
12756	T4164I	97	nsp9	C**T**DDNALAY<br>C**T**DDNALAYY	n	n	C**T**DDNALAY<br>C**T**DDNALAYY
21990	del144	62	spike	n	n	DPFLGV**Y**Y	n
22992	S477N	141	spike	n	n	n	n
23012	E484K	71	spike	n	n	TPCNGV**E**GF	n
27793	F13S	61	ORF7b	n	YLC**F**LAFLL<br>**F**LAFLLFLV<br>**F**LAFLLFLVL	n	n
29247	T325K	61	nucleopr.	n	EV**T**PSGTWL	**T**PSGTWLIY	n
29268	T332I	41	nucleopr.	n	WL**T**YTGAIKL	TPSGTWL**T**Y	n

Supplementary Table 6. Candidate CD8 T-cell epitopes with sequence alterations due to SARS CoV-2 mutations observed in the patient. nt, nucleotide position of mutation; aa ch., amino acid change (exchange or deletion); day, day when mutation became dominant (>50% of sequencing reads); n, none identified. No epitope candidates were identified for the alleles HLA-B*08:01 and HLA-C*07:01. CD8 T-cell epitope candidates were identified using anchor motifs shown in Supplementary Table 5. The table shows epitope candidates where the peptide sequence was affected by any of the 12 fixed and conserved non-synonymous SARS-CoV-2 mutations observed in the patient. All six HLA class I allotypes of the patient were considered; no epitope candidates were identified for the alleles HLA-B*08:01 and HLA-C*07:01. Pre-mutation peptide sequences are shown, sites of amino acid exchanges or deletion are underlined.

Nucleotide polymorphism	Amino acid effect	protein codon	number of sequences	count of lineages	CD8 epitope	nucleotide positions of epitope	codon positions of epitope	HLA restriction	predicted affinity to MHC (nM)	First observation (days post infection)	Presence in VOC
3del21990	S:del144	S:del144	1055144	364	undefined	undefined	undefined	undefined	undefined	50	B.1.1.7 (Alpha) B.1.525 (Eta)
G23012A	S:E484K	S:E484K	168132	245	undefined	undefined	undefined	undefined	undefined	68	P.1 (Gamma) P.2 (Zeta) P.3 (Theta) B.1.351 (Beta) B.1.525 (Eta) B.1.621 (Mu)
G22992A	S:S477N	S:S477N	66699	209	undefined	undefined	undefined	undefined	undefined	141	BA.1 (Omicron) BA.2 (Omicron) BA.3 (Omicron) BA.4 (Omicron) BA.5 (Omicron) B.1.526 (Iota)
C10116T	ORF1a:T3284I	nsp5:T21I	6284	132	undefined	undefined	undefined	undefined	undefined	134	
C12756T	ORF1a:T4164I	nsp9:T24I	3836	123	CTDDNALAY CTDDNALAYY	12752-12781	nsp9:23-32	A*01:01 A*01:01	39.7	97	
A11451G	ORF1a:Q3729R	nsp6:Q160R	9602	58	undefined	undefined	undefined	undefined	undefined	134	
G12566T	ORF1a:V4101F	nsp8:V159F	333	40	ALWEIQQVV	12545-12571	nsp8:151-160	A*02:01	7.8	20	
C29268T	N:T332I	N:T332I	273	30	TPSGTWLTY	29246-29272	N:332-340	B*35:01	4.7	20	
A9741G	ORF1a:N3159S	nsp4:N396S	58	22	undefined	undefined	undefined	undefined	undefined	141	
T27793C	ORF7b:F13S	ORF7b:F13S	83	16	undefined	undefined	undefined	undefined	undefined	50	
A4241G	ORF1a:I1326V	nsp3:I508V	99	15	undefined	undefined	undefined	undefined	undefined	50	
C29247A	N:T325K	N:T325K	20	8	TPSGTWLTY	29246-29272	N:332-340	B*35:01	4.7	61	

Supplementary Table 7: Characteristics and prevalence of non-synonymous, dominant de novo mutations. For each mutation the effect on amino acid level and the position in the corresponding SARS-CoV-2 protein is indicated. The number of sequences and the count of different lineages (Pangolin nomenclature) deposited at GISAID (accession date 17th of August 2021) that contain the respective mutation is shown. For the CD8 T-cell epitopes that have been confirmed in this study the amino acid sequence is shown together with the corresponding range of nucleotide positions and the restricting HLA allele. Predicted binding affinities to MHC are indicated based on NetMHC version 4.0 (http://www.cbs.dtu.dk/services/NetMHC/). For each mutation it is indicated if it appears in a current or deescalated variant of concern (VOC) or variant of interest (VOI) (presence in more than 85% of sequenced isolates of this lineage deposited at GISAID, accession date 19th of May 2022). MHC: major histocompatibility complex, HLA: human leukocyte antigen
del: deletion, nsp: non-structural protein.

Label Name	Description	Oligonucleotide Sequence (5’>3’)	Final Conc.
2019-nCoV_N1-F	2019-nCoV_N1 Forward Primer	GAC CCC AAA ATC AGC GAA AT	500 nM
2019-nCoV_N1-R	2019-nCoV_N1 Reverse Primer	TCT GGT TAC TGC CAG TTG AAT CTG	500 nM
2019-nCoV_N1-P	2019-nCoV_N1 Probe	FAM-ACC CCG CAT TAC GTT TGG TGG ACC-BHQ1	125 nM
2019-nCoV_N1-P	2019-nCoV_N1 Probe	FAM-ACC CCG CAT /ZEN/ TAC GTT TGG TGG ACC-3IABkFQ	125 nM

Supplementary Table 8: Used PCR-Primer and Probe sequences not commercially available targeting the Nucleocapsid as published by the CDC.

Antibody	Fluorochrome	Dilution	Clone	Vendor	Catalogue number
anti-CD69	FITC	1:5	FN50	BD Biosciences	557049
anti-CD38	PE	1:20	HIT2	BD Biosciences	555460
anti-CD4	PerCP	1:10	SK3	BD Biosciences	344624
anti-CD3	APC-H7	1:20	SK7	BD Biosciences	347340
anti-CD45	AF 700	1:100	HI30	Biolegend	304024
anti-HLA-DR	V500	1:100	G46-6	BD Biosciences	561224
anti-CD4	FITC	1:30	SK3	Biolegend	344604
anti-CD3	A700	1:15	SP34-2	BD Biosciences	557917
anti-CD8	Pacific Blue	1:30	HIT8a	Biolegend	300928

Supplementary Table 9: Details of antibodies used for flow cytometry. For each antibody the conjugated fluorochrome, the dilution for staining, clone-number, vendor and catalogue number are indicated.
