# Supplementary material for: Accumulation of mutations in antibody and CD8 T cell epitopes in a B cell depleted lymphoma patient with chronic SARS-CoV-2 infection
Source: Nat Commun. 2022 Sep 23;13:5586. doi: 10.1038/s41467-022-32772-5 (PMC9508331; doi:10.1038/s41467-022-32772-5)
Supplement: Supplementary file 3 — Description of Additional Supplementary Files [file 41467_2022_32772_MOESM3_ESM.pdf]

Description of Additional Supplementary Files

Supplementary Data 1: GISAID (https://www.gisaid.org) accession numbers for the global sequences shown in the phylogenetic tree in Fig. 2a.

We gratefully acknowledge all the authors from the originating laboratories responsible for obtaining the specimens and the submitting laboratories, where genetic sequence data were generated and shared via the GISAID Initiative, on which this research is based.
