# Supplementary material for: Accumulation of mutations in antibody and CD8 T cell epitopes in a B cell depleted lymphoma patient with chronic SARS-CoV-2 infection
Source: Nat Commun. 2022 Sep 23;13:5586. doi: 10.1038/s41467-022-32772-5 (PMC9508331; doi:10.1038/s41467-022-32772-5)
Supplement: Supplementary file 5 — Reporting Summary [file 41467_2022_32772_MOESM5_ESM.pdf]

nature portfolio

Corresponding author(s): Elham Khatamzas

Last updated by author(s): Aug 5, 2022

Reporting Summary

Nature Portfolio wishes to improve the reproducibility of the work that we publish. This form provides structure for consistency and transparency in reporting. For further information on Nature Portfolio policies, see our Editorial Policies and the Editorial Policy Checklist.

Statistics

For all statistical analyses, confirm that the following items are present in the figure legend, table legend, main text, or Methods section.

n/a	Confirmed	
☐	☒	The exact sample size (*n*) for each experimental group/condition, given as a discrete number and unit of measurement
☐	☒	A statement on whether measurements were taken from distinct samples or whether the same sample was measured repeatedly
☐	☒	The statistical test(s) used AND whether they are one- or two-sided<br>*Only common tests should be described solely by name; describe more complex techniques in the Methods section.*
☒	☐	A description of all covariates tested
☒	☐	A description of any assumptions or corrections, such as tests of normality and adjustment for multiple comparisons
☐	☒	A full description of the statistical parameters including central tendency (e.g. means) or other basic estimates (e.g. regression coefficient) AND variation (e.g. standard deviation) or associated estimates of uncertainty (e.g. confidence intervals)
☐	☒	For null hypothesis testing, the test statistic (e.g. *F*, *t*, *r*) with confidence intervals, effect sizes, degrees of freedom and *P* value noted<br>*Give P values as exact values whenever suitable.*
☒	☐	For Bayesian analysis, information on the choice of priors and Markov chain Monte Carlo settings
☒	☐	For hierarchical and complex designs, identification of the appropriate level for tests and full reporting of outcomes
☒	☐	Estimates of effect sizes (e.g. Cohen's *d*, Pearson's *r*), indicating how they were calculated

Our web collection on statistics for biologists contains articles on many of the points above.

Software and code

Policy information about availability of computer code

Data collection	Nucleic acid sequence data were collected on an Illumina MiSeq with the MiSeq Software Suite or on an Illumina HiSeq 1500 using HiSeq Control Software. ELISPOT data were acquired on a CTL ELISPOT reader. ELISA data were acquired on a Tecan multiwell plate reader using iControl software. FACS data were acquired on a CytoFlex instrument with CytExpert software or on a Becton-Dickinson Fortessa instrument with FacsDiva software. Flow cytometry FCS files were generated using this software and analyzed as described below.
Data analysis	Data analysis is outlined in detail in the supplementary material. Software packages that were used for sequence analysis are BWA v.0.7.16, freebayes v1.3.5, SnpEff 4.2, iVar v1.3, samtools 1.12-11, R 4.2, pheatmap Version 1.0.12. Molecular structures were drawn with PyMOL 2.4. Microsoft Excel 2018 for Macintosh was used for processing of ELISA and ELISPOT data. Data graphs were drawn and statistical analyses were performed with GraphPad Prism v.9.1.2. FACS data were analyzed and plots were drawn with FCSalyzer, version 2021-03. Panels were arranged into full figures with Inkscape and Adobe Illustrator. SAMBA is an acronym for "short anchor motif-based algorithm" and represents a methodological approach, not a software. MacVector was used for motif-based sequence extraction of epitope sequences from protein sequences.

For manuscripts utilizing custom algorithms or software that are central to the research but not yet described in published literature, software must be made available to editors and reviewers. We strongly encourage code deposition in a community repository (e.g. GitHub). See the Nature Portfolio guidelines for submitting code & software for further information.

Data

Policy information about availability of data

All manuscripts must include a data availability statement. This statement should provide the following information, where applicable:

- Accession codes, unique identifiers, or web links for publicly available datasets
- A description of any restrictions on data availability
- For clinical datasets or third party data, please ensure that the statement adheres to our policy

All SARS-CoV-2 sequencing data have been deposited and are available at GISAID. GISAID accession codes are indicated in Supplementary Table 3. Other data are provided within the manuscript.

Human research participants

Policy information about studies involving human research participants and Sex and Gender in Research.

Reporting on sex and gender	The patient whose case is reported was female. The five healthy SARS-CoV-2 convalescent donors were two females, three males.
Population characteristics	The patient was in her seventies. The exact age and other details are not disclosed for confidentiality reasons.<br>Healthy SARS-CoV-2 convalescent donors were in an age range from 25 to 60. The exact age is not relevant since no age-dependent conclusions about these donors are being made, and they are not presented as representative for any population group beyond their status of SARS-CoV-2 convalescent persons. The group would be too small to allow any such conclusions. Donors were chosen for inclusion in the present study due to their HLA type.
Recruitment	The main patient described in this study was recruited within the COVID-19 Registry of the LMU University Hospital Munich (CORKUM, WHO trial id DRKS00021225). Written informed consent was obtained before any study related procedure from next of kin. Clinical and routine laboratory data was prospectively collected within the COVID-19 registry and verified and complemented by individual chart review. Patient data was pseudonymized for analysis and the study was approved by the local ethics committees (No: 20-245).<br>Healthy adult convalescent donors were recruited under ethical approval (No: 17-455) of LMU University Hospital Munich. Healthy donors were recruited using local announcements of the study at LMU Klinikum and Helmholtz-Zentrum München distributed by leaflets and a public announcement board. Donors were asked to contact the study PIs for participation. Thus, donors were self-selected. Donors were not representative of the general population, since healthcare and research workers were overrepresented. No donors were recruited that were professionally or personally dependent on the principal investigators of the study. Donors were provided with a gratification of 25 Euros per blood donation. All donors provided written informed consent.
Ethics oversight	LMU University Hospital Munich (Ethikkommission bei der Medizinischen Fakultät der LMU München)

Note that full information on the approval of the study protocol must also be provided in the manuscript.

Field-specific reporting

Please select the one below that is the best fit for your research. If you are not sure, read the appropriate sections before making your selection.

☒ Life sciences ☐ Behavioural & social sciences ☐ Ecological, evolutionary & environmental sciences

For a reference copy of the document with all sections, see nature.com/documents/nr-reporting-summary-flat.pdf

Life sciences study design

All studies must disclose on these points even when the disclosure is negative.

Sample size	A single patient in her 70s, admitted to LMU-Klinikum due to COVID-19, was studied, therefore no sample size applicable.<br>Five SARS-CoV-2 convalescent donors were studied for comparison. The sample size was not determined in advance but a result of donor availability.
Data exclusions	No data were excluded.
Replication	Experiments on patient material were severely limited due to availability. Only one multimer staining experiment could be performed.<br>Clinical analyses of the patient (antibody levels, lymphocyte subsets, other clinical parameters) were performed once per time point.<br>For control donors, a total of 15 virus-specific T-cell cultures were established from five donors. Donor number was limited by availability. Six cultures could be maintained and expanded, all of these were studied in functional assays (peptide stimulation + ELISA, three to four technical replicates) twice, one result is shown. Multimer staining was performed twice each on four of these cultures, further analysis was not possible due to limited material, three representative analyses are shown. Since repeat analyses on these T-cell cultures were performed on different

	times of cultivation, numerically different but qualitatively equivalent results were expected and obtained, for each a single result is shown, and results from different time points were not averaged.<br>Virus sequencing was performed with patient samples from 21 different time points. The work was shared among two separate laboratories according to availability of machinery and personnel. 10 samples were sequenced on an Illumina MiSeq, 11 samples on an Illumina HiSeq, with two different protocols for cDNA library generation, as described in Methods.
Randomization	The single patient was chosen for this study due to the clinical and general interest of her particular case. No randomization applied as no treatments were investigated. Healthy donors were recruited based on HLA type and history of prior mild COVID-19. Covariates such as gender, background or age are not believed to influence the results of functional T cell assays that were investigated considering that all donors were healthy and had no history of immune defects. Whether or not they would harbour T cells specific for epitopes of interest for this study that could be cultivated in vitro could not be predicted due to insufficient prior literature data on expected SARS-CoV-2 immunity. Therefore this aspect of the study was of an exploratory nature, and a sample from each available HLA-matched donor from our small cohort was tested. Sample size was therefore limited by donor availability, not a priori sample size considerations.
Blinding	Only objective parameters were included in the study and therefore blinding was not applied. Regarding patient data, overall blinding was not applicable since a single patient was studied. For standardized analyses of flow cytometry and functional assays, biased analysis can be excluded.

Reporting for specific materials, systems and methods

We require information from authors about some types of materials, experimental systems and methods used in many studies. Here, indicate whether each material, system or method listed is relevant to your study. If you are not sure if a list item applies to your research, read the appropriate section before selecting a response.

Materials & experimental systems

n/a	Involved in the study	
☐	☒	Antibodies
☐	☒	Eukaryotic cell lines
☒	☐	Palaeontology and archaeology
☒	☐	Animals and other organisms
☒	☐	Clinical data
☒	☐	Dual use research of concern

Methods

n/a	Involved in the study	
☒	☐	ChIP-seq
☐	☒	Flow cytometry
☒	☐	MRI-based neuroimaging

Antibodies

Antibodies used	Phenotypic FACS analysis was performed using fresh whole blood after lysis of erythrocytes. Activation markers on T cells were evaluated with a staining panel of anti-CD45 AF700 (clone HI30, Biolegend, catalogue number 304024) and anti-CD3 APC-H7 (clone SK7, BD Biosciences, catalogue number 347340), anti-CD4 PerCP (SK3, BD Biosciences, catalogue number 344624), anti-CD69 FITC (FN50, BD Biosciences, catologue number 557049), anti-CD38 PE (HIT2, BD Biosciences, catalogue number 555460, and anti-HLA-DR V500 (G46-6, BD Biosciences, catalogue number 561224).<br>For multimer staining, antibodies were anti-CD8 Pacific Blue (HIT8a, cat. co. 300928, Biolegend), anti-CD4 FITC (SK3, cat. no. 344604, Biolegend) and anti-CD3 A700 (SP34-2, cat. no. 557917, BD Biosciences).
Validation	All antibodies were obtained from commercial vendors (Biolegend, BD Biosciences) and specificity characteristics were based on descriptions and information provided in corresponding data sheets obtained from the manufacturers.

Eukaryotic cell lines

Policy information about cell lines and Sex and Gender in Research

Cell line source(s)	Vero E6 cells used in this study were obtained from DSMZ, No. ACC 33.<br>Human T and B cell cultures were established for this study in our laboratory.
Authentication	Species-level identification was carried out by PCR targeting mitochondrial Cytochrome C Oxidase Subunit 1. Sequencing revealed Chlorocebus aethiops specific sequence.
Mycoplasma contamination	Vero E6 cells were negative for Mycoplasma contamination, tested by PCR.<br>Human T-cells were cultured for a maximum of 21 days and were not tested for mycoplasma.
Commonly misidentified lines<br>(See ICLAC register)	*Name any commonly misidentified cell lines used in the study and provide a rationale for their use.*

Flow Cytometry

Plots

Confirm that:

- [x] The axis labels state the marker and fluorochrome used (e.g. CD4-FITC).
- [x] The axis scales are clearly visible. Include numbers along axes only for bottom left plot of group (a 'group' is an analysis of identical markers).
- [x] All plots are contour plots with outliers or pseudocolor plots.
- [x] A numerical value for number of cells or percentage (with statistics) is provided.

Methodology

Sample preparation	1. PBMCs from the patient were isolated by Ficoll centrifugation, cryoconserved, thawed, and stained with multimers and antibodies within 2 hours after thawing, and immediately analyzed on a flow cytometer BD Fortessa (Fig. 2g).<br>2. Whole blood samples from the patient were collected on the day of analysis, erythrocytes were lysed, antibody staining was immediately performed, and analysis was performed on a CytoFlex LX device.<br>3. T-cell cultures were harvested and washed, stained with multimers and antibodies, and analyzed within 1 hour on a BD Fortessa.
Instrument	Beckman-Coulter CytoFlex LX (Fig. 2b); BD Fortessa (other analyses).
Software	Collection: FacsDiva (BD); analysis: FlowJo 9 (TreeStar) and FCSalyzer (Sven Mostböck, Vienna).
Cell population abundance	Cell sorting was not performed.
Gating strategy	For multimer staining, lymphocytes were gated on FSC-A vs SSC-A.<br>For phenotypic activation marker analysis, lymphocytes were gated on FSC-A vs SSC-A and subsequently on CD4 vs. CD3.

- [x] Tick this box to confirm that a figure exemplifying the gating strategy is provided in the Supplementary Information.
